# The lung microbiota in Korean patients with non-tuberculous mycobacterial pulmonary disease

**DOI:** 10.1186/s12866-021-02141-1

**Published:** 2021-03-18

**Authors:** Sung-Yoon Kang, Hyojung Kim, Sungwon Jung, Sang Min Lee, Sang Pyo Lee

**Affiliations:** 1 Division of Pulmonology and Allergy, Department of Internal Medicine, Gil Medical Center, Gachon University College of Medicine, 21, Namdong-daero 774 beon-gil, Namdong-gu, Incheon, 21565 Republic of Korea; 2 Department of Health Sciences and Technology, GAIHST, Gachon University, Incheon, Republic of Korea; 3 Department of Genome Medicine and Science, Gachon University College of Medicine, Incheon, Republic of Korea; 4 Gachon Institute of Genome Medicine and Science, Gil Medical Center, Gachon University College of Medicine, 38-13 Dokjeom-ro 3 beon-gil, Namdong-gu, Incheon, 21565 Republic of Korea

**Keywords:** Microbiome, Microbiota, Nontuberculous mycobacterium, Nontuberculous mycobacterial pulmonary disease

## Abstract

**Background:**

The microbiota of the lower respiratory tract in patients with non-tuberculous mycobacterial pulmonary disease (NTM-PD) has not been fully evaluated. We explored the role of the lung microbiota in NTM-PD by analyzing protected specimen brushing (PSB) and bronchial washing samples from patients with NTM-PD obtained using a flexible bronchoscope.

**Results:**

Bronchial washing and PSB samples from the NTM-PD group tended to have fewer OTUs and lower Chao1 richness values compared with those from the control group. In both bronchial washing and PSB samples, beta diversity was significantly lower in the NTM-PD group than in the control group (*P* = 2.25E-6 and *P* = 4.13E-4, respectively). Principal component analysis showed that the PSBs and bronchial washings exhibited similar patterns within each group but differed between the two groups. The volcano plots indicated differences in several phyla and genera between the two groups.

**Conclusions:**

The lower respiratory tract of patients with NTM-PD has a unique microbiota distribution that is low in richness/diversity.

**Supplementary Information:**

The online version contains supplementary material available at 10.1186/s12866-021-02141-1.

## Background

Nontuberculous mycobacterial (NTM) pulmonary disease (NTM-PD) is caused by pathogenic species of NTM [1–4]. NTM-PD can affect subjects of any age but is more common in those at least 50 years old, and the incidence and prevalence of NTM-PD is increasing worldwide [4–7]. However, the pathogenesis of NTM-PD is unclear, although several host and microbial factors are implicated [4, 8–12].

The microorganisms (bacteria, viruses, and fungi) residing in the human body are collectively called the microbiota [13–15]. As integral components of various organ systems, these participate in diverse cellular processes and metabolism and affect the development of various diseases. Various populations of microorganisms living in diverse cellular compartments modulate immune system function, the aberrant metabolism that triggers chronic inflammation, and cellular transformation [14–20]. However, the role of the microbiota in respiratory disease is unclear.

Two recent studies evaluated the role of the microbiota in NTM-PD [15, 21]. Sulaiman et al. collected oral wash, induced sputum and bronchoalveolar lavage samples from patients with NTM-PD and proposed that patients with NTM disease have a distinct microbial environment in the lower airways. This may be associated with components of the lower airway microbiota including taxa commonly identified as oral commensals [21]. Philley et al. assessed microbiome diversity in sputa from healthy women, women with NTM-PD, and women with both NTM-BD and breast cancer. They suggested the presence of a distinct pathogenic microbiome other than NTM [15]. However, the oral wash, sputum, and bronchial aspirate samples used in those two studies could have become contaminated by bacterial taxa in the upper airway, so they may not be representative of the microbial populations of the lower respiratory tract.

Here, we used a bronchoscopic approach to explore the role of the lung microbiota in NTM-PD by analyzing protected specimen brushing (PSB) and bronchial washing samples obtained from patients with NTM-PD in comparison with those from control subjects.

## Results

### Clinical characteristics

A total of 14 and 10 subjects were enrolled in the NTM-PD and control groups, respectively. However, three subjects in the NTM-PD group were excluded because acid-fast bacillary (AFB) cultures of bronchial washings were negative. Respiratory samples from 21 subjects (11 in the NTM-PD group and 10 in the control group) were finally analyzed. The demographic and clinical characteristics are shown in Table 1. Age, sex, smoking status, medical history, and comorbid status did not differ between the groups. BMI was significantly lower in the NTM-PD than the control group (20.4 [18.1–22.0] vs. 25.6 [17.6–31.3 kg/m^2^], *P* = 0.012). In the control group, bronchiectasis with endobronchial secretion, anthracosis, benign bronchial stenosis, and nonspecific endobronchial secretion were diagnosed after bronchoscopic examination in six (60.0%), two (20.0%), one (10.0%), and one (10.0%) subject, respectively. *Mycobacterium avium, M. intracellulare*, and *M. kansasii* were cultured from six (54.4%), three (27.3%), and one (9.1%) NTM-PD patients, respectively (Supplementary Table 1). The NTM species in an AFB culture from one patient (9.1%) could not be identified.

**Table 1:** Demographic and clinical characteristics of the study subjects

Variable	NTM-PD group (*n* = 11)	Control group (*n* = 10)	*p*-value*
Age, years	57 [23–74]	57.5 [33–83]	0.863
Female (%)	8 (72.7)	6 (60.0)	0.537
BMI (kg/m^2^)	20.4 [18.1–22.0]	25.6 [17.6–31.3]	**0.012**
Height (cm)	159.8 [152.5–183.0]	162.4 [140.0–172.0]	1.000
Weight (kg)	52.0 [45.0–63.0]	60.6 [46.4–85.1]	0.051
Smoking status (%)			0.709
Never-smoker	8 (72.7)	7 (70.0)	
Ex-smoker	1 (9.1)	2 (20.0)	
Current smoker	2 (18.2)	1 (10.0)	
Smoking level (pack-years)	35.0 [10.0–60.0]	11.4 [10.0–40.0]	0.800
Comorbidity			
Hypertension	1 (9.1)	1 (10.0)	1.000
Hyperlipidemia	1 (9.1)		1.000
Parkinson’s disease		1 (10.0)	0.476
Past medical history			
Pneumonia	1 (9.1)		1.000
Tuberculosis	1 (9.1)	2 (20.0)	1.000
Pulmonary embolism		1 (10.0)	0.476
Canine space abscess	1 (9.1)		1.000
Colon polyp	1 (9.1)		1.000
Final diagnosis after bronchoscopy (in control group)			
Bronchiectasis with endobronchial secretion		6 (60.0)	
Anthracosis		2 (20.0)	
Benign bronchial stenosis		1 (10.0)	
Endobronchial secretion		1 (10.0)	

Data are shown as medians (with ranges) or frequencies (with % values)

*NTM-PD* nontuberculous mycobacterial pulmonary disease, *BMI* body mass index

*p-values < 0.05 are shown in bold for comparisons between the NTM-PD and control groups

### RNA concentrations of the sequencing libraries

**Fig. 1:**
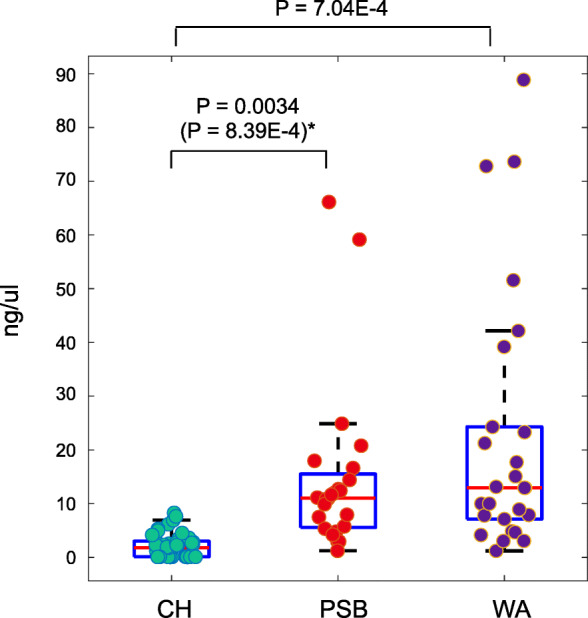
RNA concentrations in the sequencing libraries from the different samples. The concentrations are grouped by the sample acquisition protocol regardless of the group. Differences were assessed using Student’s *t*-test; *p*-values < 0.05 are shown. *P-value after discarding the two outliers with the highest concentrations from the PSB protocol. CH, bronchoscopic channel washings (negative controls); PSB, protected specimen brushing; WA, bronchial washing

Figure 1 presents the RNA concentrations of the sequencing libraries in the various samples. We excluded the possibility of environmental contamination introduced by the bronchoscopic channel by constructing 16S sequencing libraries from the negative control samples. However, as shown in Fig. 1, most of the negative control samples contained very little RNA, preventing sequencing. We thus assumed that environmental contamination was minimal during sample acquisition. Bronchial washing samples yielded libraries with higher RNA concentration compared with PSB samples.

### Richness/diversity of the NTM-PD microbiota

**Fig. 2:**
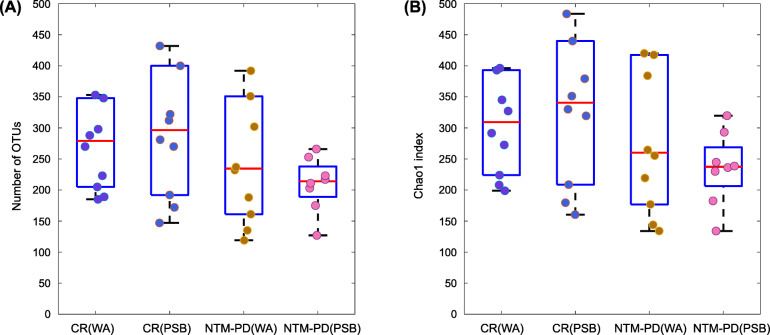
Microbial richness in bronchial washing and PSB samples from the control and NTM-PD groups. Three outlier samples (one bronchial washing and one PSB from the control group and one bronchial washing from the NTM-PD group) with over 900 OTUs and a Chao1 richness index > 1000 exist outside the plots. **a** The numbers of OTUs (a measure of microbial richness) in each group. **b** The Chao1 richness index (a measure of alpha diversity) in each group. OTU, operational taxonomic unit; CR, control; WA, bronchial washing; PSB, protected specimen brushing; NTM-PD, non-tuberculous mycobacterial pulmonary disease

**Fig. 3:**
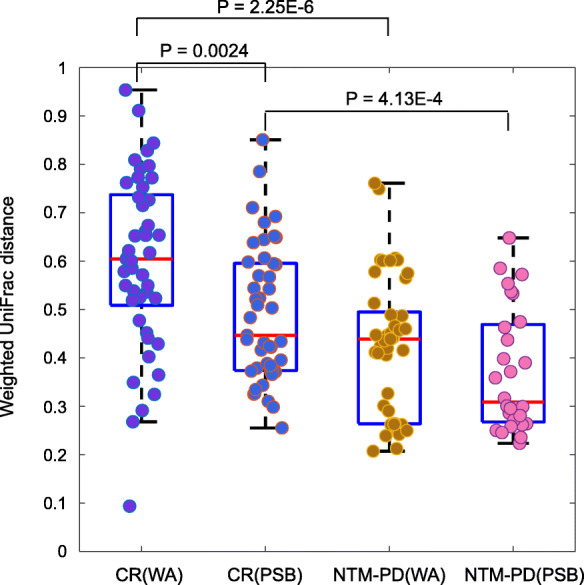
The beta diversities of each group. Each dot represents a weighted UniFrac distance between the microbial compositions of two samples in the same group. Differences were assessed using Student’s *t*-test; *p*-values < 0.05 are shown. CR, control; WA, bronchial washing; PSB, protected specimen brushing; NTM-PD, non-tuberculous mycobacterial pulmonary disease

Figure 2 shows the microbial richness and alpha diversity of each sample based on the OTU number and Chao1 richness index. NTM-PD samples had fewer OTUs (Fig. 2a) and lower Chao1 richness values (Fig. 2b) compared with the control samples, although the difference was not statistically significant. Figure 3 shows the weighted UniFrac distances between all sample pairs within each sample group, reflecting the distribution of beta diversity for each group. A higher beta diversity among samples within a group indicates greater variances in microbial community composition. Significantly lower beta diversity was observed in the NTM-PD group than in the control group in both bronchial washing (*P* = 2.25E-6) and PSB samples (*P* = 4.13E-4). In the control group, the microbiota composition had lower beta diversity in PSBs than bronchial washings (*p* = 0.0024). In the NTM-PD group, beta diversity was lower in the PSBs than bronchial washings, but the differences were not significant.

When four subjects without bronchiectasis in the control group were excluded from analysis, the beta diversity in bronchial washing and PSB samples from the NTM-PD group was significantly lower than that of the remaining six control subjects with bronchiectasis (Suppl. Fig. 1).

### Differences in microbiota composition between the NTM-PD and control groups

**Fig. 4:**
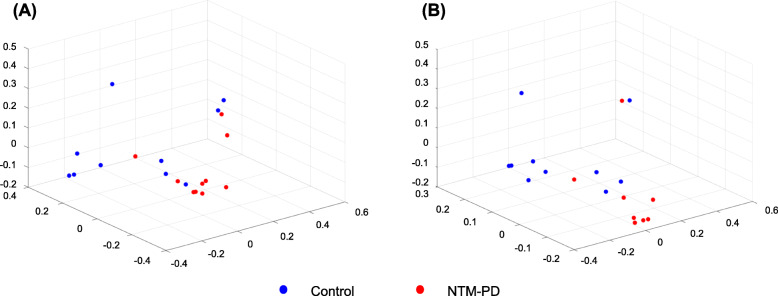
PCoA plots for the bronchial washing and PSB samples from the NTM-PD and control groups, based on weighted UniFrac distances. **a** Bronchial washing samples. **b** PSB samples. NTM-PD, non-tuberculous mycobacterial pulmonary disease; PSB, protected specimen brushing

**Fig. 5:**
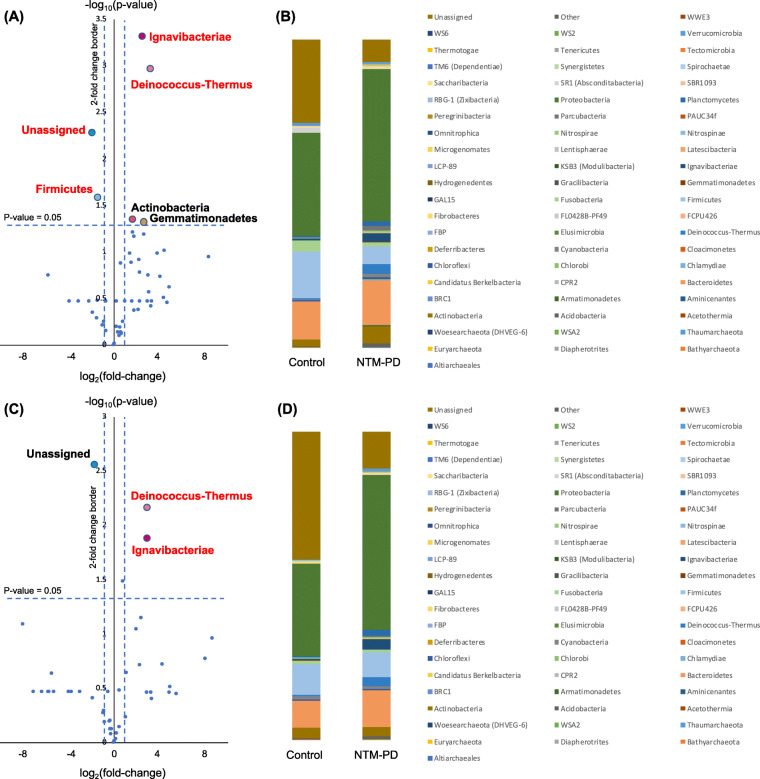
The phylum fractions in bronchial washing (**a** and **b**) and PSB (**c** and **d**) samples from the NTM-PD and control groups. The volcano plots show the relative fold changes and whether they were significant. Differences were assessed using Student’s *t*-test; the *p*-values of all phylum fractions in the NTM-PD compared with the control group are shown (**a, c**). Red indicates statistical significance (*p* < 0.05) after adjustment for BMI (by ANCOVA). NTM-PD, non-tuberculous mycobacterial pulmonary disease; PSB, protected specimen brushing; BMI, body mass index

The PCoA plots for bronchial washings (Fig. 4a) and PSBs (Fig. 4b) were similar within each group. In the NTM-PD group, the bronchial washing and PSB samples were clustered closely, and most could be distinguished from those of the control group, the patterns of which were disperse. The proportions of individual phyla in bronchial washings varied (Fig. 5a and b). Compared with the control group, the proportions of *Ignavibacteriae, Deinococcus–Thermus, Actinobacteria,* and *Gemmatimonadetes* were increased (2.67 vs. 0.48%, 3.16 vs. 0.36%, 5.61 vs. 2.18%, and 0.19 vs. 0.03%, respectively), and those of *Firmicutes* and unassigned fractions were decreased (5.84 vs. 15.27% and 7.16 vs. 27.06%, respectively) in NTM-PD bronchial washings. After adjustment for BMI, the increase in the proportions of *Ignavibacteriae* and *Deinococcus–Thermus,* and the decreases in proportions of *Firmicutes* and unassigned fractions remained significant. The PSBs yielded similar results (Fig. 5c and d), with minor changes in a few phyla (*Firmicutes*, *Actinobacteria*, and *Gemmatimonadetes*). After adjustment for BMI, the increases in proportions of *Deinococcus–Thermus* and *Ignavibacteriae* remained significant. When four subjects without bronchiectasis in the control group were excluded from the analysis, the proportions of *Ignavibacteriae, Deinococcus–Thermus,* and *Gemmatimonadetes* were higher (2.67 vs. 0.25%, 3.16 vs. 0.09%, and 0.19 vs. 0.00%, respectively) in the NTM-PD bronchial washing samples compared to samples from the remaining six control subjects with bronchiectasis. By contrast, the proportions of *Actinobacteria and Firmicutes* were not different between the NTM-PD group and control subjects with bronchiectasis (Suppl. Fig. 2A). In PSB samples from the NTM-PD group, the proportions of *Deinococcus–Thermus* and *Ignavibacteriae* were higher (7.16 vs. 0.60% and 5.84 vs. 0.54%, respectively) compared to the remaining six control subjects with bronchiectasis (Suppl. Fig. 2B).

**Fig. 6:**
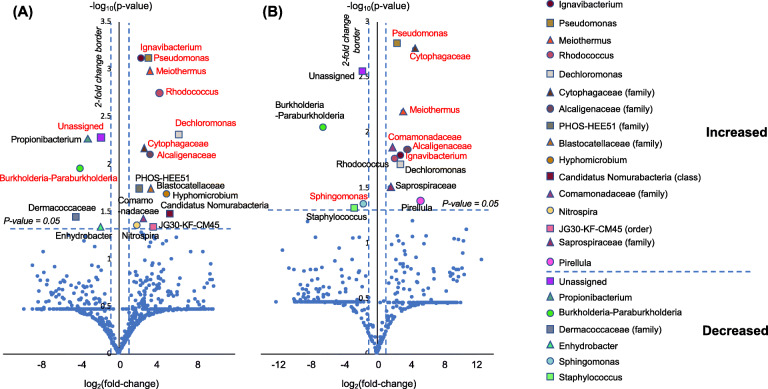
Comparisons of genus (or higher-level OTU classifications in the absence of genus-level taxonomic information) in bronchial washing and PSB samples from the NTM-PD and control groups. **a** Bronchial washing samples. **b** PSB samples. The volcano plots show the relative fold changes and whether they were significant. Differences were assessed using Student’s *t*-test; the *p*-values for each genus (or higher-level OTU in the absence of genus-level taxonomic data) represent the statistical significance of abundance changes in the NTM-PD group compared with the control group. Red indicates statistical significance (*p* < 0.05) after adjustment for BMI (by ANCOVA). NTM-PD, non-tuberculous mycobacterial pulmonary disease; PSB, protected specimen brushing; BMI, body mass index

**Fig. 7:**
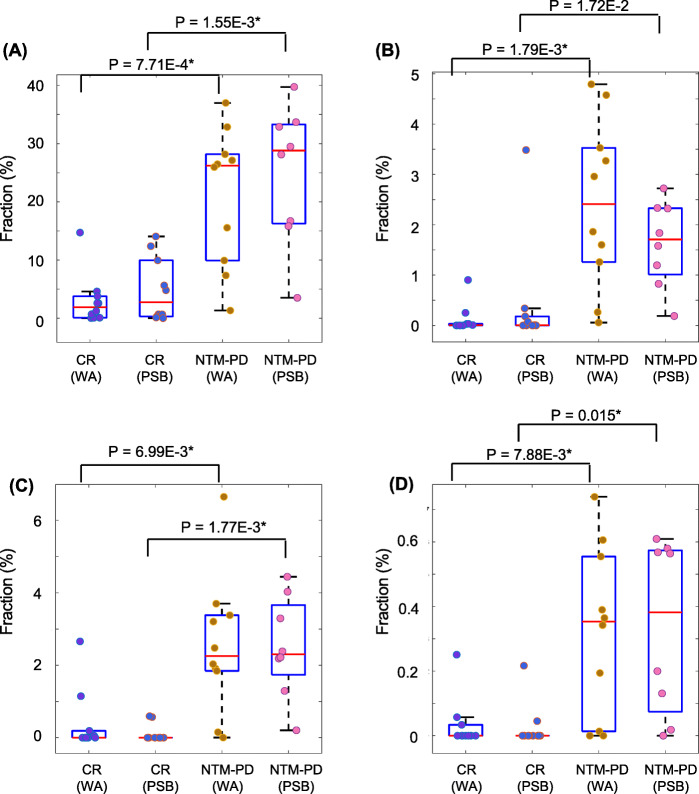
The genera and families exhibiting significant increases in bronchial washing and PSB samples from the NTM-PD compared with control groups. Differences were assessed using Student’s *t*-test; the *p*-values are shown if *p* < 0.05. **a** *Pseudomonas*, **b** *Rhodococcus*, **c** Cytophagaceae, and **d** Alcaligenaceae. *, *P* < 0.05 after adjustment for BMI (by ANCOVA); NTM-PD, non-tuberculous mycobacterial pulmonary disease; PSB, protected specimen brushing; BMI, body mass index

The microbiota fractions at the genus level (or higher level OTU classifications in the absence of genus-level taxonomic information) in bronchial washings and PSBs were compared between the NTM-PD and control groups (Fig. 6). The proportions of various genera differed between the groups in bronchial washings and PSBs. In bronchial washings, the proportions of *Ignavibacterium, Pseudomonas, Meiothermus, Rhodococcus, Dechloromonas, Cytophagaceae* family*, Alcaligenaceae* family, Phos-Hee51 family*, Blastocatellaceae* family, *Hyphomicrobium, Candidatus Nomurabacteria* classes*, Comamonadaceae* family*,* and *Nitrospira* and JG30-KF-CM45 orders were increased, whereas those of *Propionibacterium, Bukholderia–Paraburkholderia, Dermacoccaceae, Enhydrobacter*, and unassigned fractions were decreased in the NTM-PD compared with control groups. After adjustment for BMI, the increase in the proportions of *Ignavibacterium, Pseudomonas, Meiothermus, Rhodococcus, Dechloromonas, Cytophagaceae* family*,* and *Alcaligenaceae* family*,* and the decreases in the proportions of *Bukholderia–Paraburkholderia,* and unassigned fractions remained significant. In the PSB samples, the proportions of *Pseudomonas, Cytophagaceae* family*, Meiothermus, Comamonadaceae* family*, Alcaligenaceae* family*, Ignavibacterium, Rhodococcus, Dechloromonas, Saprospiraceae*, and *Pirellula* were increased, whereas those of *Bukholderia–Paraburkholderia, Sphingomonas, Staphylococcus*, and unassigned fractions were decreased in the NTM-PD group compared with the control group. After adjustment for BMI, the increases in the proportions of *Pseudomonas, Cytophagaceae* family*, Meiothermus, Comamonadaceae* family*, Alcaligenaceae* family*, and Ignavibacterium,* and the decrease in the proportion of *Sphingomonas* remained significant. The proportions of *Pseudomonas, Rhodococcus, Cytophagaceae,* and *Alcaligenaceae* were consistently higher in bronchial washings and PSBs from the NTM-PD group compared with the control group (Fig. 7). With the exception of the proportion of *Rhodococcus* in bronchial washing samples, the differences in the proportions of the four microbes remained significant after adjustment for BMI. When four control subjects without bronchiectasis were excluded from the analysis, the proportions of *Ignavibacterium, Meiothermus, Pseudomonas, Rhodococcus, Dechloromonas, Alcaligenaceae* family, Phos-Hee51 family*, Nitrospira*, *Blastocatellaceae* family, *Cytophagaceae* family*, Hyphomicrobium, Candidatus Nomurabacteria* classes*,* JG30-KF-CM45 orders, and *Comamonadaceae* family were still greater, and those of *Bukholderia–Paraburkholderia, Propionibacterium,* and unassigned fractions were lower in the NTM-PD bronchial washing samples compared to samples from the remaining six control subjects with bronchiectasis. By contrast, the proportions of *Dermacoccaceae* and *Enhydrobacter* did not decreased (Suppl. Fig. 3A). In PSB samples from the NTM-PD group, *Cytophagaceae* family*, Pseudomonas, Meiothermus, Comamonadaceae* family, *Ignavibacterium, Alcaligenaceae* family*,* and *Pirellula* increased compared to samples from the remaining six control subjects with bronchiectasis. By contrast, the proportions of *Rhodococcus, Dechloromonas, Saprospiraceae, Bukholderia–Paraburkholderia, Sphingomonas, Staphylococcus,* and unassigned fractions did not differ between the two groups (Suppl. Fig. 3B).

## Discussion

Patients with NTM-PD provided bronchial washing and PSB samples with lower microbial richness as well as non-significant lower alpha diversity. The significantly lower beta diversity observed in bronchial washing and PSB samples from the NTM-PD group indicates smaller variances in microbial community composition compared to the control group. Beta diversity was also significantly lower in bronchial and PSB samples from the NTM-PD group when only six control subjects with bronchiectasis were analyzed, Therefore, NTM-PD, but not bronchiectasis, is associated with a homogenous microbial community composition. The two sampling approaches yielded similar microbiome distributions, but the PSB samples from the NTM-PD group exhibited lower alpha and beta diversities than did bronchial washing samples. In the bronchial washing and PSB samples from the NTM-PD group, some phyla and genera increased, while others decreased compared to the control group and the control subgroup with bronchiectasis. These differences remained significant after adjustment for BMI.

Respiratory samples (especially PSBs) from NTM-PD patients tended to exhibit lower alpha diversities compared with those from control subjects. In a previous study, the alpha diversities of sputum samples from females with NTM-PD and those with both NTM-PD and breast cancer were significantly lower than those of samples from healthy females, consistent with our results [15]. In another study, the alpha diversities in oral washes, sputum, and BAL samples did not differ in NTM-positive versus -negative sputum cultures [21]. However, in this study, oral washings exhibited higher alpha diversities than those of sputum, which in turn exhibited a bacterial load of approximately 100-fold greater than that of BAL samples. Furthermore, PSB samples from the NTM-PD groups exhibit a non-significantly lower alpha diversity compared to bronchial washing samples from both groups and PSB samples from the control group.

In our study and those of others, most subjects with NTM-PD were slender postmenopausal women, indicating that a lean body and a lack of estradiol are associated with development of NTM-PD [21–23]. Estradiol protects against NTM infection. Indeed, ovariectomized mice had a higher NTM burden in the lung at 3–6 weeks after infection compared to sham-operated mice and E2-treated ovariectomized mice [24]. Moreover, long-term E2 administration in ovariectomized mice increased IL-1β secretion and inducible nitric oxide synthase expression, promoting the killing of intracellular mycobacteria by macrophages [25]. Slender individuals with NTM-PD exhibited abnormal expression of not only IFN-γ and IL-12, which inhibit the development of NTM-PD [5], but also leptin and adiponectin, two important immune modulating adipokines secreted by fat tissue [26]. Adipocytes are also reported to regulate the expression of IL-12 and receptors in the presence of inflammation [27]. Collectively, the lack of estradiol, and adipose tissue and the resulting elevated levels of proinflammatory mediators impair resistance to colonization by external microbes, thus leading to loss-of-containment of pathogens and consequently mycobacterial infection. and this process may be similar to that of *Mycobacterium tuberculosis* [28]. In our study, this vulnerability to colonization by external microbes in patients with NTM-PD may explain the elevated richness of *Pseudomonas, Rhodococcus, Comamonadaceae, Nitrospiraceae,* and *Cytophagaceae* in lower airway samples, which, like NTM, are abundant in groundwater and treated water [29–31]. Chronic and intermittent *Pseudomonas* infection is associated with NTM infection [32], and *Rhodococcus* and other species of *Actinomycetales* are frequently identified in humans or animals with confirmed or suspected mycobacterial infection [33–38]. Coinfection with these microbes may lead to the establishment of homogenous microbial communities in patients with NTM-PD, who showed lower within-group diversity (beta diversity) than the control subjects in this study.

Our work had several limitations. First, as we did not evaluate upper airway samples (oral washes and sputum), we do not know whether the upper airway microbiome differed between the groups. Second, we did not measure the levels of inflammatory biomarkers (such as cytokines) or determine neutrophil or lymphocyte counts. Therefore, it is unclear whether communities of microbes contribute to the buildup of *inflammation* and whether microbial products or metabolites lead to the development of respiratory diseases. Further research using biomarkers or other cellular markers is needed to elucidate the association between inflammation and the microbiome composition in NTM diseases. Finally, we found that 16S rRNA sequencing identified mycobacteria in only 54.5% of bronchial washing samples and 27.3% of PSB samples from NTM-PD patients (Supplementary Table 2). Similarly, 16S rRNA sequencing identified NTM in only 47% of BAL samples from subjects whose sputum was positive for NTM cultures despite use of a nested mycobacterial microbiome approach. Thus, 16S rRNA gene sequencing is not adequately sensitive, and technical improvements are needed [21, 39].

Despite these limitations, our study had certain merits. We compared bronchial washings and PSBs within subjects. PCoA revealed that the microbiome distributions were similar, suggesting that both bronchial washings and PSBs reflect the lower airway status of NTM-PD patients. The PSB samples exhibited the lowest alpha and beta diversities and lacked oral commensals such as *Firmicutes,* thus optimally reflecting lower airway status. Also, several microbes differed between the NTM-PD and control groups. Patients with NTM-PD have a distinct lower-airway environment, which may be associated with microbial taxa thought of as environmental. These data suggest a connection between the pathogenesis of NTM-PD and colonization by environmental microbes, which warrants further mechanistic studies.

## Conclusion

Lower respiratory samples from NTM-PD patients exhibited a unique microbiome that was less rich/diverse compared to that of the control subjects, with some phyla and genera being increasing in abundance, and others decreasing. Further studies are required to verify these findings and to determine whether those microbes interact (positively or negatively) with NTM to provoke inflammation and/or antibiotic resistance independent of the NTM-PD status.

## Methods

### Study subjects and sample collection

Patients who underwent bronchoscopy due to a suspicion of NTM-PD on chest CT in a 1600-bed tertiary university medical center in Incheon, Republic of Korea between August 2017 and August 2018 were prospectively enrolled in the NTM-PD group after providing written informed consent. NTM-PD was suspected when nodular bronchiectatic lesions typical of NTM-PD were observed on chest computed tomography [40–42]. Patients who underwent bronchoscopy to examine suspicious endobronchial lesions that were not typical of NTM-PD, tuberculosis, malignancy, or any known disease or condition of the lower respiratory tract except bronchiectasis were enrolled in the control group. The exclusion criteria were malignancy at any site; an infection or serious disease of the neural, cardiovascular, renal, hepatobiliary, gastrointestinal, hematological, or respiratory system; use of any antibiotic during the prior month; perceived vulnerability; and refusal to participate. All patients underwent bronchoscopy, and we collected PSB and bronchial washing samples. Prior to bronchoscopy, all subjects received topical anesthesia (lidocaine delivered via a nebulizer) and were sedated with midazolam and fentanyl. The bronchoscopic channels were washed with 5 mL of sterile 0.9% (w/v) saline (negative control samples). In the NTM-PD group, respiratory specimens were collected from lesional bronchi using a protected brush; each brush was chopped into small pieces (using a sterile wire cutter), suspended in 5 mL of sterile 0.9% (w/v) saline, and vortexed. Lesional bronchi were also washed with 5 mL of sterile saline. In the control group, PSB and bronchial washing samples were collected from random bronchi. We recorded the subjects’ demographic and clinical characteristics including data on age, sex, height, weight, body mass index (BMI), smoking status and level, comorbidities, and medical history.

### DNA extraction

DNA was extracted from respiratory specimens on the day of bronchoscopy using the PowerSoil DNA Isolation Kit from Mo Bio Laboratories Inc. (Carlsbad, CA) according to the manufacturer’s instructions (manual ver. 07272016).

### PCR and sequencing

The V3–4 regions of bacterial 16S rRNA genes were amplified using PCR. PCR was conducted in reaction mixture with a total volume of 25-μL containing 2.5 μL of DNA extract, 12.5 μL of KAPA HiFi Hotstart readyMix (Kapa Biosystems, Boston, MA), 5.0 μL of 1 M forward primer (5′- TCGTCGGCAGCGTCAGATGTGTATAAGAGACAGCCTACGGGNGGCWGCAG-3′), and 5.0 μL of 1 M reverse primer (5′ -GTCTCGTGGGCTCGGAGATGTGTATAAGAGACAGGACTACHVGGGTATCTAATCC-3′). PCR comprised in initial denaturation at 95 °C for 3 min, followed by 25 cycles of denaturation at 95 °C for 30 s, annealing at 51 °C for 30 s, and extension at 72 °C for 30 s, followed by a final extension at 72 °C for 5 min. Template size distributions were explored using the Agilent Technologies 2100 Bioanalyzer (Agilent, Palo Alto, CA) fitted with a DNA 1000 chip. The library was sequenced from both ends using the Illumina MiSeq sequencer based at Macrogen (Seoul, Republic of Korea). FASTQ files were generated from the base-calls using the Illumina software package bcl2fastq.

### Sequence analysis and microbial diversity

The analytical tools of QIIME ver. 1.9.1 were used to process sequence data and perform taxonomic analysis [43]. Paired reads were merged using FLASH ver. 1.2.11 (minimum overlap 10 bp; maximum overlap 100 bp; maximum allowed mismatch:overlap length ratio 0.25) [44]. Chimera detection and operational taxonomic unit (OTU) clustering were performed using the cd-hit-dup and cd-hit-otu programs of CD-HIT ver. 4.5.4 [45]. Sequences were aligned using the parallel_align_seqs_pynast.py script and taxonomic assignment conducted using the assign_taxonomy.py script and the UCLUST method [46]. SILVA ver.128 served as the reference database for sequence alignment and taxonomic assignment [47]. The Chao1 richness index was evaluated using QIIME, which yields both the alpha and beta diversities (the latter was calculated using the weighted UniFrac distances). We employed QIME to assess dissimilarity among the samples. UniFrac is a phylogenetic distance metric used to compare phylogenetic distances among different samples [15]. We performed principal coordinate analysis (PCoA) to plot these phylogenetic metrics [15].

### Statistical analysis

Continuous variables were compared by the *t*-test or analysis of covariance (ANCOVA), and frequencies were compared by Fisher’s exact test. A *p-*value < 0.05 was considered indicative of statistical significance. False discovery rate (FDR) adjustment of *p*-values was not conducted due to the small number of samples. Statistical analysis was performed using MATLAB ver. R2019a (MathWorks, Natick, MA).

### Ethics statement

The study protocol was reviewed and approved by the Institutional Review Board of Gachon University Gil Medical Center (IRB approval number: GAIRB2017–065), Incheon, Republic of Korea. The study was registered at clinicaltrials.gov (no. NCT04079400).

## Supplementary Information

**Additional file 1: Table S1.** NTM species cultured from the NTM-PD group. NTM, nontuberculous *Mycobacterium*; NTM-PD, non-tuberculous mycobacterial pulmonary disease.

**Additional file 2: Table S2.** Rate of *Mycobacterium* identification by 16S rRNA sequencing.

**Additional file 3: Figure S1.** Beta diversities. In the control group, four subjects without bronchiectasis were excluded from the analysis. Dots represents the weighted UniFrac distance between the microbial compositions of two samples in the same group. Differences were assessed using Student’s *t*-test; the *p*-values are shown if *p* < 0.05. CR, control; WA, bronchial washing; PSB, protected specimen brushing; NTM-PD, non-tuberculous mycobacterial pulmonary disease.

**Additional file 4: Figure S2.** Phylum fractions in bronchial washing (A) and PSB (B) samples from the NTM-PD group and six control subjects with bronchiectasis. The volcano plots show relative fold changes and their significance. Differences were assessed using Student’s *t*-test; the *p*-values from the comparisons between all phylum fractions in the NTM-PD group and the six control subjects with bronchiectasis are shown. NTM-PD, non-tuberculous mycobacterial pulmonary disease; PSB, protected specimen brushing.

**Additional file 5: Figure S3.** Genera (or higher-level OTU classifications in the absence of genus-level taxonomic information) in bronchial washing and PSB samples from the NTM-PD group and six control subjects with bronchiectasis. (A) Bronchial washing samples. (B) PSB samples. Volcano plots show relative fold changes and their significance. Differences were assessed using Student’s *t*-test; the *p*-values for each genus (or higher-level OTU) represent the significance of abundance changes in the NTM-PD group compared with six control subjects with bronchiectasis. NTM-PD, non-tuberculous mycobacterial pulmonary disease; PSB, protected specimen brushing; OTU, operational taxonomic unit.

## Data Availability

Analysis script: https://www.sysbiolab.org/respiratory-analysis The datasets used and/or analyzed during the current study available from the corresponding author on reasonable request.
