# Supplementary material for: The lung microbiota in Korean patients with non-tuberculous mycobacterial pulmonary disease
Source: BMC Microbiol. 2021 Mar 18;21:84. doi: 10.1186/s12866-021-02141-1 (PMC7977250; doi:10.1186/s12866-021-02141-1)
Supplement: Supplementary file 1 — Additional file 1: Table S1. NTM species cultured from the NTM-PD group. NTM, nontuberculous Mycobacterium; NTM-PD, non-tuberculous mycobacterial pulmonary disease. [file 12866_2021_2141_MOESM1_ESM.docx]

**Supplementary Table 1.** NTM species cultured from the NTM-PD group.

| Species | Frequency (%) |
| --- | --- |
| *Mycobacterium avium*  *M. intracellulare*  *M. kansasii*  Unidentified | 6 (54.5)  3 (27.3)  1 (9.1)  1 (9.1) |

NTM, nontuberculous *Mycobacterium*; NTM-PD, nontuberculous mycobacterial pulmonary disease.
