# Supplementary material for: The lung microbiota in Korean patients with non-tuberculous mycobacterial pulmonary disease
Source: BMC Microbiol. 2021 Mar 18;21:84. doi: 10.1186/s12866-021-02141-1 (PMC7977250; doi:10.1186/s12866-021-02141-1)
Supplement: Supplementary file 2 — Additional file 2: Table S2. Rate of Mycobacterium identification by 16S rRNA sequencing. [file 12866_2021_2141_MOESM2_ESM.docx]

**Supplementary Table 2.** Rate of *Mycobacterium* identification by 16S rRNA sequencing.

| Sample | All subjects (*n*=21) | NTM-PD group  (n=11) | Control group (n=10) | *p*-value* |
| --- | --- | --- | --- | --- |
| Bronchial washing | 7 (33.3) | 6 (54.5) | 1 (10.0) | **0.029** |
| PSB | 4 (19.0) | 3 (27.3) | 1 (10.0) | 0.206 |

Data are shown as frequencies (% value). **p*-values <0.05 are shown in bold for comparisons between the NTM-PD and control groups. NTM-PD, nontuberculous mycobacterial pulmonary disease; PSB, protected specimen brushing.
