# Supplementary figures and images for: The lung microbiota in Korean patients with non-tuberculous mycobacterial pulmonary disease
Source: BMC Microbiol. 2021 Mar 18;21:84. doi: 10.1186/s12866-021-02141-1 (PMC7977250; doi:10.1186/s12866-021-02141-1)

## Slide 1
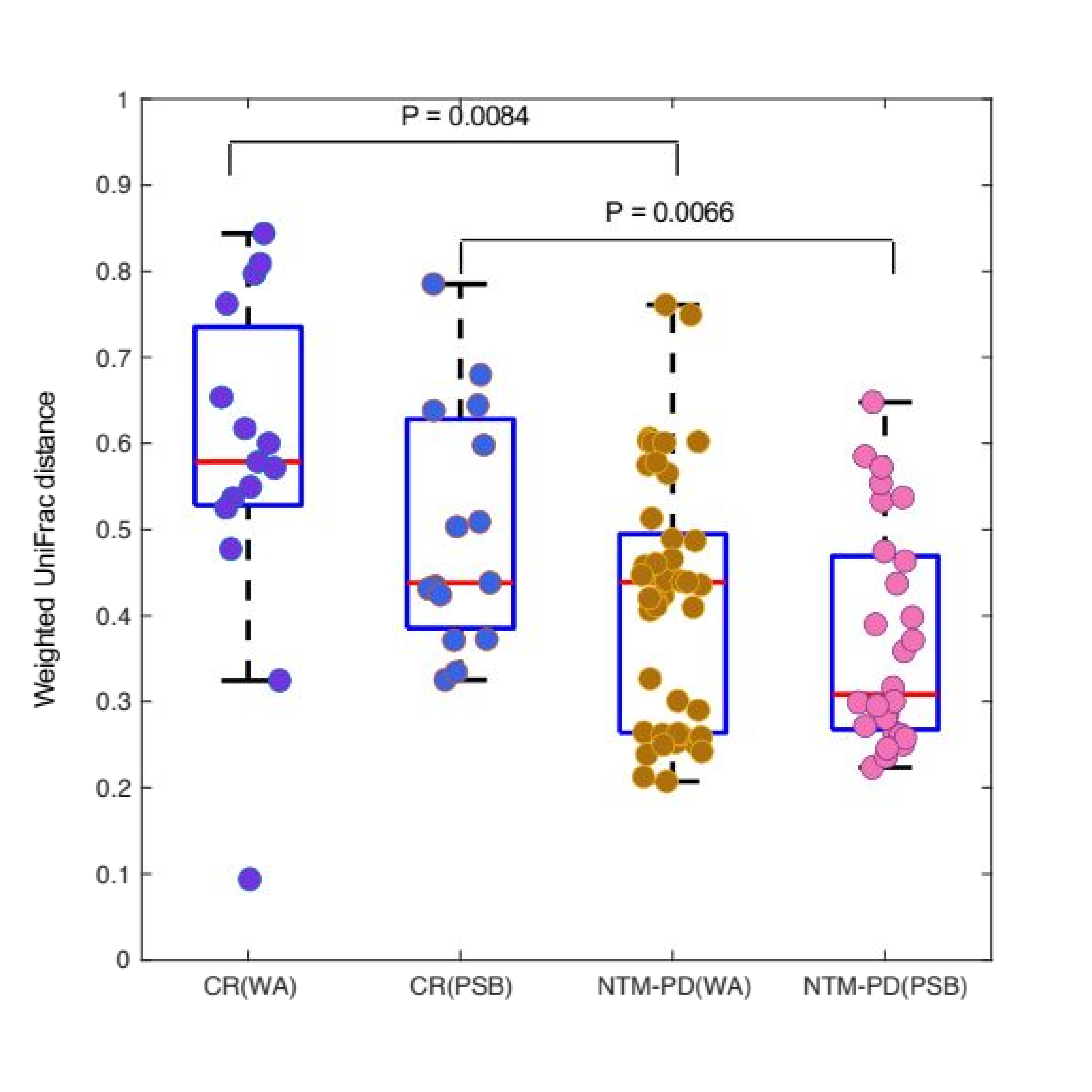

Supplement: Supplementary file 3 — Additional file 3: Figure S1. Beta diversities. In the control group, four subjects without bronchiectasis were excluded from the analysis. Dots represents the weighted UniFrac distance between the microbial compositions of two samples in the same group. Differences were assessed using Student’s t-test; the p-values are shown if p < 0.05. CR, control; WA, bronchial washing; PSB, protected specimen brushing; NTM-PD, non-tuberculous mycobacterial pulmonary disease. [file 12866_2021_2141_MOESM3_ESM.pptx]

## Slide 1
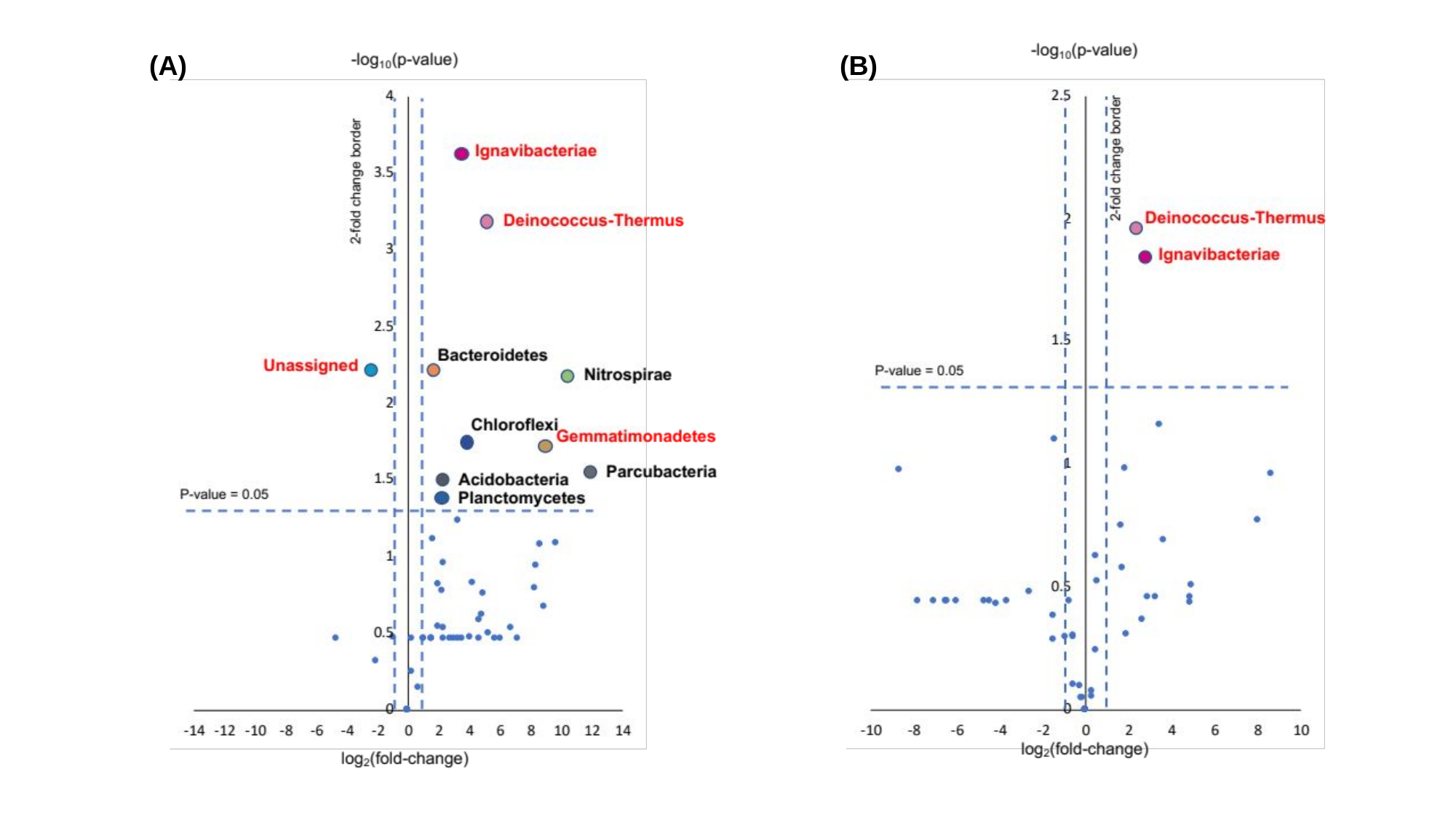

(A)
(B)

Supplement: Supplementary file 4 — Additional file 4: Figure S2. Phylum fractions in bronchial washing (A) and PSB (B) samples from the NTM-PD group and six control subjects with bronchiectasis. The volcano plots show relative fold changes and their significance. Differences were assessed using Student’s t-test; the p-values from the comparisons between all phylum fractions in the NTM-PD group and the six control subjects with bronchiectasis are shown. NTM-PD, non-tuberculous mycobacterial pulmonary disease; PSB, protected specimen brushing. [file 12866_2021_2141_MOESM4_ESM.pptx]

## Slide 1
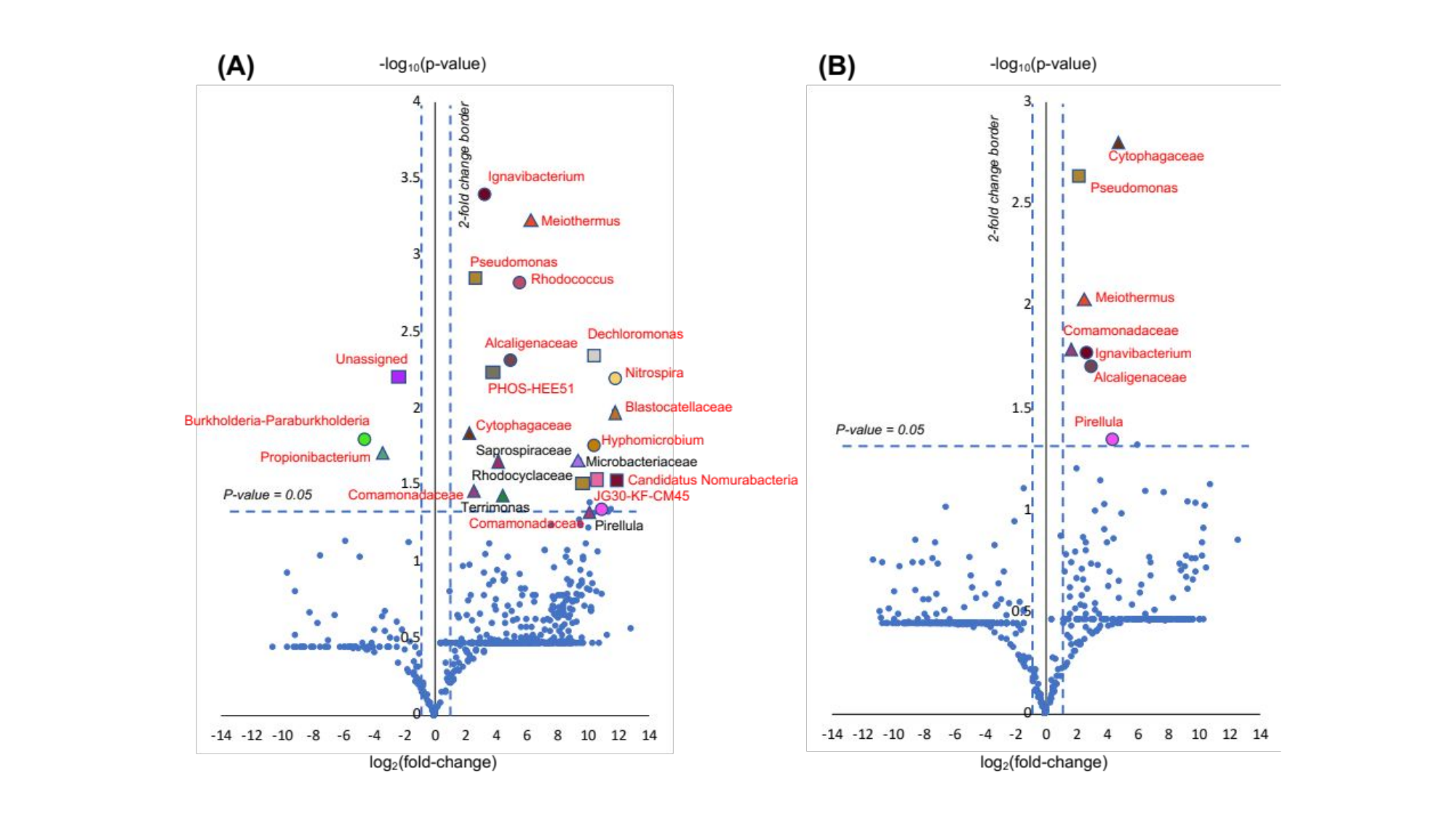

Supplement: Supplementary file 5 — Additional file 5: Figure S3. Genera (or higher-level OTU classifications in the absence of genus-level taxonomic information) in bronchial washing and PSB samples from the NTM-PD group and six control subjects with bronchiectasis. (A) Bronchial washing samples. (B) PSB samples. Volcano plots show relative fold changes and their significance. Differences were assessed using Student’s t-test; the p-values for each genus (or higher-level OTU) represent the significance of abundance changes in the NTM-PD group compared with six control subjects with bronchiectasis. NTM-PD, non-tuberculous mycobacterial pulmonary disease; PSB, protected specimen brushing; OTU, operational taxonomic unit. [file 12866_2021_2141_MOESM5_ESM.pptx]
